# Draft Genome Sequence of *Acetobacterium bakii* DSM 8239, a Potential Psychrophilic Chemical Producer through Syngas Fermentation

**DOI:** 10.1128/genomeA.01070-15

**Published:** 2015-09-24

**Authors:** Soonkyu Hwang, Yoseb Song, Byung-Kwan Cho

**Affiliations:** a Department of Biological Sciences and KAIST Institute for the BioCentury, Korea Advanced Institute of Science and Technology, Daejeon, South Korea; b Intelligent Synthetic Biology Center, Daejeon, South Korea

## Abstract

*Acetobacterium bakii* DSM 8239 is an anaerobic, psychrophilic, and chemolithoautotrophic bacterium that is a potential platform for producing commodity chemicals from syngas fermentation. We report here the draft genome sequence of *A. bakii* DSM 8239 (4.14 Mb) to elucidate its physiological and metabolic properties related to syngas fermentation.

## GENOME ANNOUNCEMENT

Anaerobic acetogenic bacteria convert syngas, such as CO_2_ with H_2_ or CO, via the reductive acetyl-coenzyme A (CoA) pathway to acetate as a main product (1). Its autotrophic metabolism can be used for producing biofuels and valuable chemicals with higher catalyst specificity, lower energy cost, and less catalyst poisoning than those with conventional methods (2–6). However, the use of syngas as carbon and energy sources for the bacteria is often limited due to the low solubility of syngas in the liquid phase. For instance, the increasing solubility of hydrogen resulted in increased growth and acetate production by the mesophilic acetogenic bacterium *Acetobacterium woodii* (7). Thus, with its high growth rate at low temperatures, *Acetobacterium bakii* is a potential microbial platform of syngas fermentation (8). In this study, we determined the draft genome sequence of *A. bakii* DSM 8239 to understand its physiological and metabolic properties related to syngas fermentation at low temperature.

*A. bakii* was cultured under anaerobic conditions at 20°C in DSMZ medium 135 supplemented with 5 g/liter fructose (8). Genomic DNA was isolated using the PowerSoil DNA isolation kit (Mo Bio Laboratories, Carlsbad, CA), followed by fragmentation using Covaris S220 (Covaris, Inc., Woburn, MA). Fragmented genomic DNA was used to construct the Illumina paired-end library using a TruSeq kit (Illumina, Inc., San Diego, CA), and then sequenced using a MiSeq version 2 instrument with the 2 × 150-cycle paired-end read recipe. CLC Genomics Workbench (CLC bio, Aarhus, Denmark) was used to trim the obtained reads using the default parameter, resulting in 8,044,838 reads with an average read length of 139.97 bp. The genome was assembled using the *de novo* program in CLC (minimum contig length, 1,051; automatic bubble size, yes; word size, auto; perform scaffolding, yes). Following assembly, the Rapid Annotations using Subsystems Technology server (9) was used to annotate the draft genome sequence. rRNA and tRNA genes were predicted using RNAmmer 1.2 (10) and tRNAscan-SE 1.31 (11), respectively.

The *A. bakii* draft genome sequence is 4,141,442 bases with 91 contigs, with 41.2% G+C content, 4,024 predicted open reading frames, 48 tRNA genes, and 5 rRNA genes. The assembled genome contains the Wood-Ljungdahl pathway for converting CO_2_ to acetyl-CoA. Distinct from other sequenced mesophilic acetogen genomes, *A. bakii* contains a two-component system for membrane fluidity regulation genes, including an autophosphorylatable histidine kinase and a DNA binding response regulator, which activate or regulate the gene expression of membrane lipid composition at different temperatures (12). *A. bakii* contains a metabolic pathway that produces ethanol from pyruvate by the pyruvate decarboxylase (EC 4.1.1.1), alcohol dehydrogenase (EC 1.1.1.1), and propionaldehyde synthesis pathways, producing 1-propanol. Further study of the draft genome sequence will enable *A. bakii* to be used as an industrial producer for syngas fermentation.

### Nucleotide sequence accession numbers.

The draft genome sequence of *A. bakii* DSM 8239 has been deposited in the DDBJ/EMBL/GenBank database under the accession no. LGYO00000000. The version described in this paper is the first version, LGYO00000000.1.

## References

[1] Sattley WM, Madigan MT 2007 Cold-active acetogenic bacteria from surficial sediments of perennially ice-covered Lake Fryxell, Antarctica. Microbiol Lett 272:48–54. doi:10.1111/j.1574-6968.2007.00737.x. 17456187

[2] Bredwell MD, Srivastava P, Worden RM 1999 Reactor design issues for synthesis-Gas fermentations. Biotechnol Prog 15:834–844. doi:10.1021/bp990108m. 10514253

[3] Schiel-Bengelsdorf B, Dürre P 2012 Pathway engineering and synthetic biology using acetogens. FEBS Lett 586:2191–2198. doi:10.1016/j.febslet.2012.04.043. 22710156

[4] Song Y, Jeong Y, Shin HS, Cho B- 2014 Draft genome sequence of *Clostridium scatologenes* ATCC 25775, a chemolithoautotrophic acetogenic bacterium producing 3-methylindole and 4-Methylphenol. Genome Announc 2(3):e00459-14. doi:10.1128/genomeA.00459-14. PMC4022816 24831152

[5] Song Y, Cho BK 2015 Draft genome sequence of chemolithoautotrophic acetogenic butanol-producing *Eubacterium limosum* ATCC 8486. Genome Announc 3(1):e01564-14. doi:10.1128/genomeA.01564-14. PMC4333668 25676768

[6] Song Y, Hwang S, Cho B 2015 Draft genome sequence of *Clostridium aceticum* DSM 1496, a potential butanol producer through syngas fermentation. Genome Announc 3(2):e00258-15. doi:10.1128/genomeA.00258-15. PMC4417690 25931594

[7] Demler M, Weuster-Botz D 2011 Reaction engineering analysis of hydrogenotrophic production of acetic acid by *Acetobacterium woodii*. Biotechnol Bioeng 108:470–474. doi:10.1002/bit.22935. 20830677

[8] Kotsyurbenko OR, Simankova MV, Nozhevnikova AN, Zhilina TN, Bolotina NP, Lysenko AM, Osipov GA 1995 New species of psychrophilic acetogens: *Acetobacterium bakii* sp. nov., *A. paludosum* sp. nov., *A. fimetarium* sp. nov. Arch Microbiol 163:29–34. doi:10.1007/BF00262200.

[9] Aziz RK, Bartels D, Best AA, DeJongh M, Disz T, Edwards RA, Formsma K, Gerdes S, Glass EM, Kubal M, Meyer F, Olsen GJ, Olson R, Osterman AL, Overbeek RA, McNeil LK, Paarmann D, Paczian T, Parrello B, Pusch GD, Reich C, Stevens R, Vassieva O, Vonstein V, Wilke A, Zagnitko O 2008 The RAST server: Rapid Annotations using Subsystems Technology. BMC Genomics 9:75. doi:10.1186/1471-2164-9-75. 18261238 PMC2265698

[10] Lagesen K, Hallin P, Rodland EA, Staerfeldt H-H, Rognes T, Ussery DW 2007 RNAmmer: consistent and rapid annotation of ribosomal RNA genes. Nucleic Acids Res 35:3100–3108. doi:10.1093/nar/gkm160. 17452365 PMC1888812

[11] Lowe TM, Eddy SR 1997 tRNAscan-SE: a program for improved detection of transfer RNA genes in genomic sequence. Nucleic Acids Res 25:955–964. doi:10.1093/nar/25.5.0955. 9023104 PMC146525

[12] Cybulski LE, Albanesi D, Mansilla MC, Altabe S, Aguilar PS, de Mendoza D 2002 Mechanism of membrane fluidity optimization: isothermal control of the *Bacillus subtilis* acyl-lipid desaturase. Mol Microbiol 45:1379–1388. doi:10.1046/j.1365-2958.2002.03103.x. 12207704

